# Macrophage activation markers are associated with infection and mortality in patients with acute liver failure

**DOI:** 10.1111/liv.15928

**Published:** 2024-04-08

**Authors:** Anna Cavazza, Evangelos Triantafyllou, Roberto Savoldelli, Salma Mujib, Ellen Jerome, Francesca M. Trovato, Florent Artru, Roosey Sheth, Xiao Hong Huang, Yun Ma, Francesco Dazzi, Tasneem Pirani, Charalambos G. Antoniades, William M. Lee, Mark J. McPhail, Constantine J. Karvellas, the US Acute Liver Failure Study Group

**Affiliations:** ^1^ Department of Inflammation Biology, School of Inflammation and Microbial Science, Institute of Liver Studies King's College London London UK; ^2^ Liver Intensive Therapy Unit Institute of Liver Studies, King's College Hospital London UK; ^3^ Section of Hepatology and Gastroenterology, Department of Metabolism, Digestion and Reproduction Imperial College London London UK; ^4^ School of Cardiovascular and Metabolic Medicine and Science King's College London London UK; ^5^ Division of Digestive and Liver Diseases UT Southwestern Medical Center Dallas Texas USA; ^6^ Division of Gastroenterology (Liver Unit), Department of Critical Care Medicine University of Alberta Edmonton Canada

**Keywords:** acute liver failure, innate immunity, macrophages, organ failure, sepsis

## Abstract

**Background and Aims:**

Acute liver failure is a multisystem disorder with a high mortality and frequent need for emergency liver transplantation. Following massive innate immune system activation, soluble markers of macrophage activation are released during liver damage and their association with disease severity and prognosis requires exploration.

**Methods:**

Patients ALF from the United States Acute Liver Failure Study Group (USALFSG, *n* = 224) and King's College Hospital (*n* = 40) together with healthy controls (HC, *n* = 50) were recruited. Serum from early (Days 1–3) and late (>Day 3) time points were analysed for MAMs by enzyme‐linked immunosorbent assay correlated to markers of illness severity and 21‐day spontaneous survival. Surface expression phenotyping was performed via Flow Cytometry on CD14^+^ monocytes.

**Results:**

All MAMs serum concentrations were significantly higher in ALF compared to controls (*p* < .0001). sCD206 concentration was higher in early and late stages of the disease in patients with bacteraemia (*p* = .002) and infection in general (*p* = .006). In MELD‐adjusted multivariate modelling, sCD206 and sCD163 were independently associated with mortality. CD14^+^ monocyte expression of CD206 (*p* < .001) was higher in patients with ALF compared with controls and correlated with SOFA score (*p* = .018). sCD206 was independently validated as a predictor of infection in an external cohort.

**Conclusions:**

sCD206 is increased in serum of ALF patients with infections and poor outcome and is upregulated on CD14^+^ monocytes. Later measurements of sCD163 and sCD206 during the evolution of ALF have potential as mechanistic predictors of mortality. sCD206 should be explored as a biomarker of sepsis and mortality in ALF.

**List of abbreviations**
ALF: acute liver failure
ALI: acute liver injury
APAP: acetaminophen
ARLD: alcohol‐related liver disease
CVVH: continuous veno‐venous hemofiltration
DAMP: damage‐associated molecular patterns
DILI: drug‐induced liver injury
ELISA: enzyme‐linked immunosorbent assay
HC: healthy control
HE: hepatic encephalopathy
HLA‐DR: histocompatibility class‐II antigen
ICP: intracerebral pressure
KC: Kupffer cells
KCC: King's College Criteria
LT: liver transplantation
MAM: marker of macrophage activation
MELD: model for end‐stage liver disease
MerTK: Mer‐tyrosine kinase
miRNA: microRNA
OPN: Osteopontin
PAMP: pathogen‐associated molecular patterns
PBMC: peripheral blood mononuclear cells
PD‐1: programmed cell death protein‐1
PD‐L1: programmed death ligand‐1
ROC: receiver operating characteristic
RTT: renal replacement therapy
sCD163: soluble form of CD163
SLPI: secretory leukocyte protease inhibitor
sMR/CD206: soluble mannose receptor
SOFA: Sequential Organ Failure Assessment
sPD‐L1: soluble PD‐L1
TFS: transplant‐free survival
USALFG: United States Acute Liver Failure Study Group

**Key points**

- Acute liver failure is a life‐threatening disease occurring in people without pre‐existing liver disease.
- They require admission to intensive care and often need a liver transplant.
- One of the main causes of death is the occurrence of infection due to a defect in the immune system's defence. This paper is exploring several markers of activation of immune cells called macrophages.
- These can be measured directly in the blood and may be used in the future as a signal of impending infection or death.

## 1 INTRODUCTION

Acute liver failure (ALF) is a rare life‐threatening multisystem disorder characterised by hepatic encephalopathy (HE) and coagulopathy in the absence of pre‐existing liver disease,^1^ with a high mortality and need for emergency liver transplantation (LT). Patients are prone to septic complications^2^, ^3^ and underlying monocyte–macrophage activation abnormalities are strongly implicated in this predisposition.^4^

There is an initial activation of hepatic inflammatory cells and monocyte recruitment to the liver from the peripheral blood, with increased local immune responses and vascular permeability. Kupffer cells (KC) are also activated by binding to and phagocytosing pathogen‐associated molecular patterns (PAMP) and damage‐associated molecular patterns (DAMP).^4^, ^5^ Subsequently, there is an immune response shift that promotes hepatic regeneration and fosters a pro‐restorative microenvironment in the liver. Nevertheless, this alteration results in a state of immune paresis.^6^ Circulating markers of monocyte/macrophage activation (MAMs) are released during liver damage and resolution and may be associated with disease severity and prognosis.^7^

Several molecules are implicated and have been studied in the last decade, including secretory leukocyte protease inhibitor (SLPI), a mediator of anti‐inflammatory responses^8^ and CD163, a scavenger receptor expressed mainly on macrophages. Its soluble form (sCD163) is released from activated macrophages and high concentrations (>26 mg/L) are associated with fatal outcome and contributes to the pathogenesis in ALF.^9^, ^10^

Mer tyrosine kinase (MerTK) and programmed cell death 1 (PD‐1) expression by monocytes and macrophages contributes to downregulation of innate immune responses in acute liver injury,^11^ inflammation and cancer.^12^ We recently demonstrated that immune cell membrane‐bound PD‐1, programmed death ligand 1 (PD‐L1) and soluble (sPD‐L1) contribute to the suppression of KC and monocyte antimicrobial responses after acute liver injury.^13^

Osteopontin (OPN) is involved in numerous pathological conditions including inflammation and is a chemoattractant for macrophages and neutrophils during injury in liver diseases.^14^ Hepatic expression and serum concentrations of OPN are elevated in patients with alcohol‐related hepatitis and alcohol‐related liver disease (ARLD).^15^, ^16^

Soluble mannose receptor (sMR/CD206), found on macrophages and dendritic cells and released during liver damage, has been associated with liver disease severity^17^ and prognosis in community acquired pneumonia. The presence of CD206^+^ macrophages was a feature of fatal pneumonia cases.^18^

Despite this evidence, these molecules have not been well characterised as markers of poor outcome in large multi‐aetiological populations. Given the clinical unmet need to improve prognostication and transplant indication,^19^ assessing MAMs in large ALF cohorts with appropriate validation is warranted.

We hypothesised that serum MAMs are associated with mortality and the development of infection in ALF and conducted a nested case–control study using samples from prospectively enrolled patients from the Acute Liver Failure Study Group (USALFSG) registry. We further validated any findings in a cohort from King's College Hospital.

## 2 METHODS

### 2.1 Participants

Inclusion criteria were as follows: (1) evidence of ALF according to the enrolment criteria of the US ALFSG (see operational definitions in Supplementary materials), (2) participant age ≥ 18 years and (3) primary diagnosis of ALF as determined by the site investigator and further adjudicated by an external review committee. Exclusion criteria were as follows: (1) evidence of cirrhosis/acute‐on‐chronic liver failure.

### 2.2 Clinical variables and endpoints

The ALFSG registry (data coordinating centre at Medical University of South Carolina, Department of Public Health Sciences, Charleston, South Carolina) contains prospectively collected demographic, clinical (Days 1–7), biochemical (Days 1–7) and outcome data from patients enrolled between 1998 and 2014. The primary endpoint for this study was a 21‐day transplant‐free survival (TFS). Demographic, clinical, laboratory and outcome data were recorded prospectively at both early (Days 1–3) and late (Day >3) time points simultaneous to blood sampling. Data assessed in this study included age, sex, laboratory data (full blood count, creatinine, liver function tests, INR, ammonia, lactate and arterial pH) and requirement for organ support (mechanical ventilation, vasopressors and renal replacement therapy). Prognostic scores including the King's College criteria (KCC),^19^ model for end‐stage liver disease (MELD) and Sequential Organ Failure Assessment (SOFA) scores were calculated from these data. The primary outcome was a 21‐day survival. A validation cohort of patients was recruited at King's College Hospital in London between June 2020 and August 2023 with the same criteria, samples were used for flow cytometry and validation studies. Healthy volunteers (HC) were recruited as controls.

### 2.3 Enzyme‐linked immunosorbent assay

The following assays were used according to the manufacturers' instructions: Human SLPI Quantikine ELISA (R&D Systems; DP100), Human CD163 Duoset ELISA (R&D Systems; DY1607‐05), Human OPN ELISA (Bio‐Techne; DY1433), Human MerTK kit (Bio‐Techne; DYC891‐5), Human MMR (CD206) ELISA (Insight Biotechnology; ELH‐MMR) and Human PD‐L1 ELISA (ThermoFisher; BMS2212 and BMS2212TEN).

### 2.4 Flow cytometry

HC and ALF patients from King's College Hospital were recruited as part of the ‘Immunometabolism in Sepsis, Inflammation and Liver Failure Syndromes/IMET’ (North West Haydock Research Ethics Committee No.: 19/NW/0750, IRAS No.: 244089) studies within 48 h of admission. For those lacking capacity, consultees provided written informed consent. Clinical and laboratory data were collected entered in a database. Blood was collected into lithium heparin Vacutainers (BD, Franklin Lakes, NJ) and peripheral blood mononuclear cells (PBMCs) isolated (see Supplementary Methods). PBMCs were thawed after having been isolated by density gradient centrifugation using Ficoll‐Paque Plus (GE Healthcare, UK).

Flow cytometry gating strategy and analysis are described in the Supplementary Methods.

### 2.5 Statistical analysis

Continuous data were described with median (range) and Mann–Whitney *U*‐tests were used to compare groups. Categorical variables were described as frequencies and groups were compared with chi‐squared tests. When comparing three groups, Kruskal–Wallis tests was used with post hoc testing for false discovery. Correlation was by Spearman's method. Receiver operating characteristic (ROC) curve analysis was performed and *p*‐values were generated using the DeLong method. Data were analysed using SPSS (IBM SPSS Statistics, version 26.0, IBM Corp., Armonk, NY, USA), MedCalc (MedCal Software, version 19.1.5, Ostend, Belgium) and GraphPad Prism (version 8.1.1, GraphPad Software, La Jolla, CA USA).

## 3 RESULTS

### 3.1 Patient characteristics

Two hundred and twenty‐four patients (79 (36%) male) were studied from the USALFSG, with a median (range) age of 42 (17–81). Four hundred and sixty‐six (466) samples, including ALF and acute liver injury (ALI) patients, were analysed. Thirty‐four healthy controls (16 (47%) male) with a median (range) age of 29 (20–45) were also recruited. Forty ALF patients (median age 36, 17 (43%) male) and 16 HC (median age 29.7, 8 (53%) male) were recruited as validation cohort at King's College Hospital in London. This included both peripheral blood serum and PBMCs.

In the discovery cohort, 87 ALF cases (39%) had drug‐induced liver injury (DILI), 46 (20%) indeterminate ALF, 60 (27%) with APAP‐ALF, 15 (7%) autoimmune and 16 (7%) for other aetiologies such as vascular or hypoxic hepatitis. The median admission MELD score was 32 (8–59) and 76 out of 224 (34%) patients spontaneously survived, 76 (34%) died and 72 (32%) underwent liver transplantation. Twenty‐nine patients and 15 continuous veno‐venous hemofiltration (CVVH). Ninety‐five patients had high‐grade HE and 16 had an intracerebral pressure (ICP) bolt inserted.

In the validation cohort, 27 patients had DILI, among which 20 had APAP‐ALF. Median admission MELD score was 37.5 (10–50), 25 patients spontaneously survived and 6 were transplanted. Fifteen patients had positive cultures, 30 required RRT in form of CVVH and 24 had high‐grade HE, no ICP bolt was used.

### 3.2 Markers of monocyte/macrophage activation are upregulated in the serum of patients with acute liver failure compared to healthy controls

**FIGURE 1:**
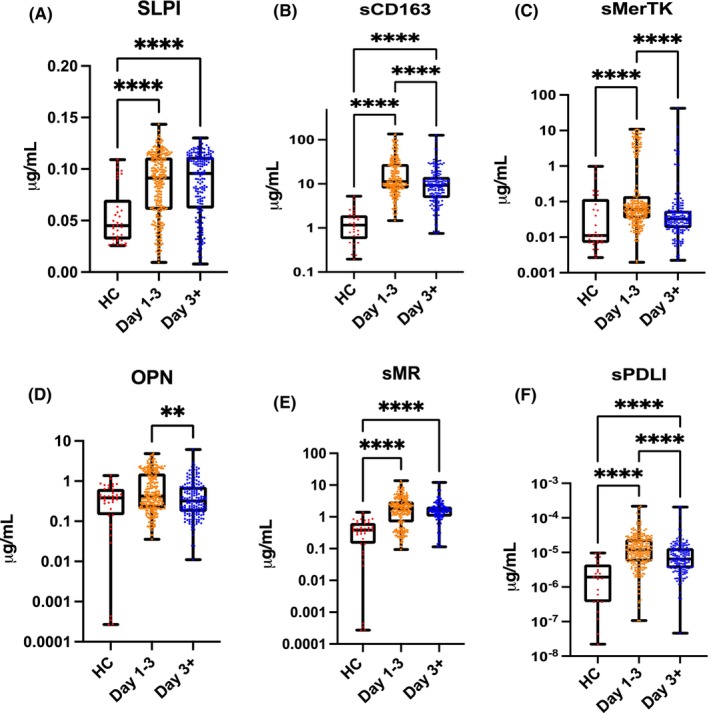
Macrophage activation markers in the serum of patients with acute liver failure (ALF) measured by enzyme‐linked immunosorbent assay (ELISA). Six molecules were measured from the cohort of patients with ALF in the acute liver failure study group early (Days 1–3 following admission to hospital) and late (Day 3+) in the course of disease and healthy controls (HC) from volunteers at King's College Hospital. (A, E) Secretory leukocyte peptidase inhibitor (SLPI) and soluble macrophage mannose receptor (sMR) concentrations are higher in both early and late groups compared to HC. (B, F) soluble cluster of differentiation 163 (sCD163) and soluble programmed death ligand 1 (sPD‐L1) concentrations are higher ALF (both groups) compared to HC and higher in early compared to late groups. (C) Soluble Mer tyrosine kinase (sMerTK) concentrations are higher in the early group compared to HC and to the late group. (D) Osteopontin (OPN) concentrations are higher in the early group compared to the late one. *p < .05, **p < .01, ***p < .001, ****p < .0001.

SLPI concentrations were higher in ALF compared to HC (Figure 1A) and remained unaltered during admission. Conversely, patients showed increased sMerTK and sCD163 levels at Day 1 of recruitment (*p* < .0001) (Figure 1B,C) falling dynamically after Day 3 of admission (for both *p* < .0001). OPN (Figure 1D) (*p* < .05), sMR (Figure 1E) (*p* < .0001) and sPD‐L1 (Figure 1F) concentrations were significantly increased in ALF compared to controls (*p* < .0001) (Table 1).

**TABLE 1:** Macrophage activation markers from healthy controls and the cohort of patients with ALF, ALI in the acute liver failure study group measured early (Days 1–3) and late (Day 3+) during admission. Categorical variables are given as *n* (%) and continuous as median (range).

	Macrophage activation marker	Healthy control	ALF (all)	ALF (D/T)	ALF (S)	ALI	*p‐*value
EARLY Days 1–3	SLPI (10^−3^ μg/mL)	45.06 (25.77–109.11)	90.78 (9.46–143.34)	91.12 (9.46–137.52)	91.52 (20.52–143.34)	84 (23.53–129.43)	<.001
	sCD163 (μg/mL)	1.16 (.20–5.23)	11.00 (1.50–135.00)	11.22 (1.98–134.66)	10.780 (1.47–98.39)	9.388 (2.08–39.75)	<.001
	sMerTK (10^−3^ μg /mL)	11.15 (2.66–988.56)	60.94 (1.96–10827.28)	66.85 (12.50–10827.28)	41.52 (1.96–8147.67)	44.08 (11.35–3500.37)	<.001
	OPN (10^−3^ μg /mL)	13.82 (.00–67.78)	438.88 (35.71–4842.13)	347.38 (35.71–4461.79)	721.61 (45.46–4842.13)	231.25 (40.60–2546.63)	<.001
	sMR (10^−3^ μg/mL)	380.78 (.27–1382.22)	1798.49 (93.65–13727.50)	2027.614 (116.87–13727.50)	1268.42 (93.65–8671.67)	2152.56 (300.86–8041.04)	<.001
	sPDL1 (10^−6^ μg/mL)	.19 (.00–9.70)	10.93 (.00–218.3)	10.93 (.00–218.3)	13.61 (.00–89.24)	7.318 (.77–47.52)	<.001
LATE Day 3+	SLPI (10^−3^ μg/mL)	45.06 (25.77–109.11)	95.70 (7.88–130.02)	95.31 (7.88–128.12)	96.19 (27.45–130.02)	72.65 (24.24–125.42)	<.001
	sCD163 (μg/mL)	1.16 (.19–5.23)	9.20 (.74–126.52)	10.24 (.75–12.65)	7.70 (.78–61.65)	8.01 (1.69–62.95)	<.001
	sMerTK (10^−3^ μg/mL)	11.15 (2.66–988.56)	32.63 (2.22–42183.52)	38.44 (2.22–42183.52)	24.62 (2.87–4889.48)	32.24 (3.75–11332.15)	.009
	OPN (10^−3^ μg/mL)	13.82 (.00–67.78)	321.40 (11.04–6087.39)	306.55 (11.04–6087.39)	323.67 (24.83–2295.45)	184.35 (20.48–1347.66)	<.001
	sMR (10^−3^ μg/mL)	380.78 (.27–1382.22)	1560.90 (113.75–12,088.63)	1600.30 (113.75–12,088.63)	1464.88 (139.31–6510.28)	1551.80 (178.14–5752.40)	<.001
	sPDL1 (10^−6^ μg/mL)	.19 (.00–9.70)	5.83 (.00–206.8)	6.66 (.00–206.8)	4.829 (.00–121.6)	4.41 (.00–28.66)	<.001

### 3.3 Markers of monocyte/macrophage activation are upregulated in the serum of patients with acute liver failure compared to those with acute liver injury

Serum sCD163, OPN and sPD‐L1 concentrations were higher in ALF patients compared to those with ALI, whereas there were no statistically significant differences between ALI and ALF patients for SLPI, sMerTK and sMR/sCD206 (Figures S1 and S2).

### 3.4 Aetiology is an important factor in determining upregulation of markers of monocyte/macrophage activation

**FIGURE 2:**
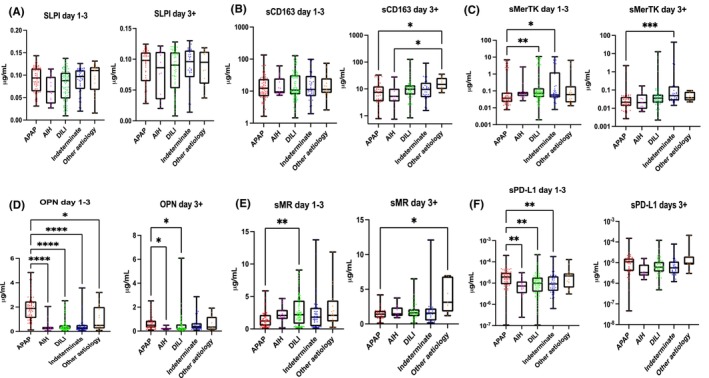
Macrophage activation markers in the serum of patients with acute liver failure (ALF) measured by enzyme‐linked immunosorbent assay (ELISA) early (Days 1–3) and late (Day3+) during admission. Aetiologies of ALF considered were acetaminophen (APAP), autoimmune hepatitis (AIH), drug‐induced liver injury (DILI), indeterminate and other aetiology (vascular, ischaemic). (A) SLPI is lowest in patients with autoimmune hepatitis compared to APAP in the early stages of admission while there is no significant difference between different aetiologies in the late course of the disease. (B, C) sCD163 and sMerTK are lower in APAP compared to other aetiologies only in late samples. sMerTK is higher in DILI and indeterminate causes compared to APAP in early and late stages. (D) OPN is higher in patients with APAP compared to autoimmune hepatitis, DILI, indeterminate and other aetiologies in the early stages. The significance is maintained after the third day of admission with higher values in APAP compared to AIH and DILI. (E) sMR is higher in DILI in early stages and in other aetiologies later during admission compared to APAP. (F) sPD‐L1 levels are lower in autoimmune hepatitis, DILI and indeterminate aetiologies compared to those with APAP in early stage of disease. **p < .01, ***p < .001, ****p < .0001.

In our sub‐analysis (Figure 2), APAP overdose was taken as the reference aetiology. Serum SLPI concentration was lowest in patients with autoimmune hepatitis (*p* ≤ .05) compared to APAP in the early stages (Days 1–3) of admission while there was no significant difference between different aetiologies in the later (Day 3+) course of the disease (Figure 2A). sMerTK and sCD163 (only in late samples) concentrations were lower in APAP compared to other aetiologies (vascular, ischaemic) (*p* ≤ .05). sMerTK concentration was higher in DILI and indeterminate causes compared to APAP in early and late stages (early *p* ≤ .01, *p* ≤ .05; late *p* ≤ .05, *p* ≤ .0001) (Figure 2B,C). In the first 3 days of admission, OPN concentration was higher in patients with APAP compared to autoimmune hepatitis (*p* < .0001), DILI (*p* < .0001), indeterminate (*p* < .0001) and other aetiologies (*p* ≤ .05). The difference was maintained after the third day of admission with higher values in APAP compared to AIH and DILI (*p* ≤ .05; *p* ≤ .01) (Figure 2D). sMR concentration was higher in DILI in the early stages (*p* ≤ .001) and in other aetiologies (vascular, ischaemic) later during admission compared to APAP. sMR concentration was higher in DILI in early stages and in other aetiologies later during admission compared to APAP (Figure 2E). sPD‐L1 levels are lower in autoimmune hepatitis, DILI and indeterminate aetiologies compared to those with APAP in early stage of disease (Figure 2F).

### 3.5 Relationship between markers of monocyte/macrophage activation and infection

**FIGURE 3:**
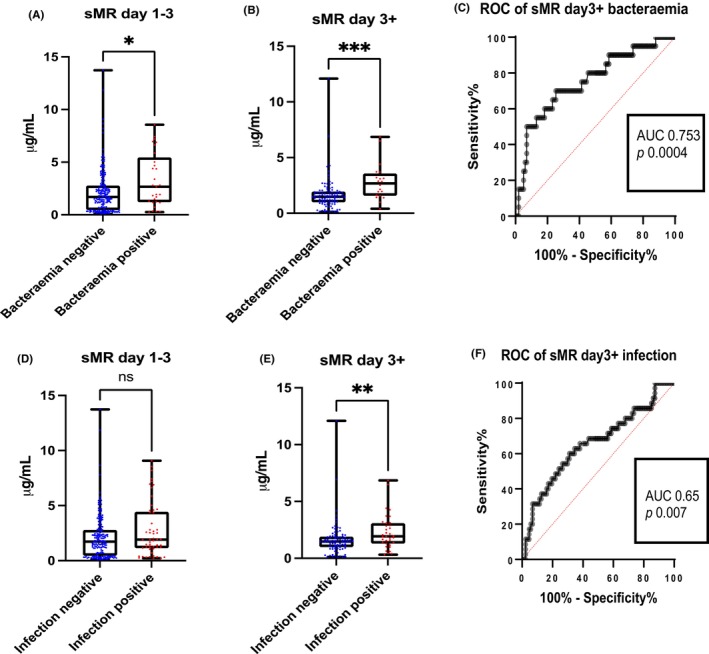
Comparison of macrophage activation markers depending on transplantation status measured by enzyme‐linked immunosorbent assay (ELISA). (A, D, F) Secretoty leukocyte peptidase inhibitor (SLPI), Osteopontin (OPN) and soluble programmed death ligand 1 (sPD‐L1) concentrations are lower in those patients with acute liver failure (ALF) who were transplanted compared to those who survived and died. (C) Soluble Mer tyrosine kinase (sMerTK) concentrations are higher in those ALF who died compared to those who survived. (E) Soluble macrophage mannose receptor (sMR) concentrations are higher in those transplanted ALF compared to those who survived. **p < 0.01***p < 0.001****p < 0.0001.

In the first 3 days of admission, ALF patients with positive bacteraemia showed increased sMR concentration compared to those with negative blood culture (*p* = .022, AUROC = .63, CI .51–.74) (Figure 3A). The same trend was seen after the third day of admission, when both sMR and sPD‐L1 concentrations were higher in patients with positive bacteraemia compared to those with negative blood culture (respectively *p* = .0002, AUROC = .75, CI .63–.87; *p* = .041, AUROC = .63, CI .51–.74) (Figure 3B; Figure S10E,F). In the late (Day 3+) but not early stage of disease, both sMR and sPD‐L1 concentrations were higher in patients with infection compared to those without (respectively *p*= .006, AUROC = .65, CI .54–.76; *p* = .041, AUROC = .60, CI .51–.69) (Figure 3E,F; Figure S13E,F).

### 3.6 Markers of monocyte/macrophage activation are correlated with model for end‐stage liver disease

Most of the MAMs were positively correlated with MELD at both early and late time points, SLPI (*r* = .18, *p* = .0057; *r* = .19, *p* = .0254), sCD163 (*r* = .21, *p* = .0031; *r* = .39, *p* < .0001), sMerTK (*r* = .23, *p* = .0005; *r* = .40, *p* < .0001), OPN (*r* = .2, *p* = .0011; *r* = .2, *p* = .007) and sPDL1 (*r* = .27, *p* < .0001; *r* = .32, *p* = .0001). sMR had no significant correlation with MELD at early (*r* = .07, *p* = .25) or late (*r* = .09, *p* = .32) stages (Figure S3A,B). Only serum sMerTK concentration was higher in KCC‐positive patients (Figures S8 and S9).

### 3.7 Markers of monocyte/macrophage activation are associated with mortality in patients with acute liver failure

**FIGURE 4:**
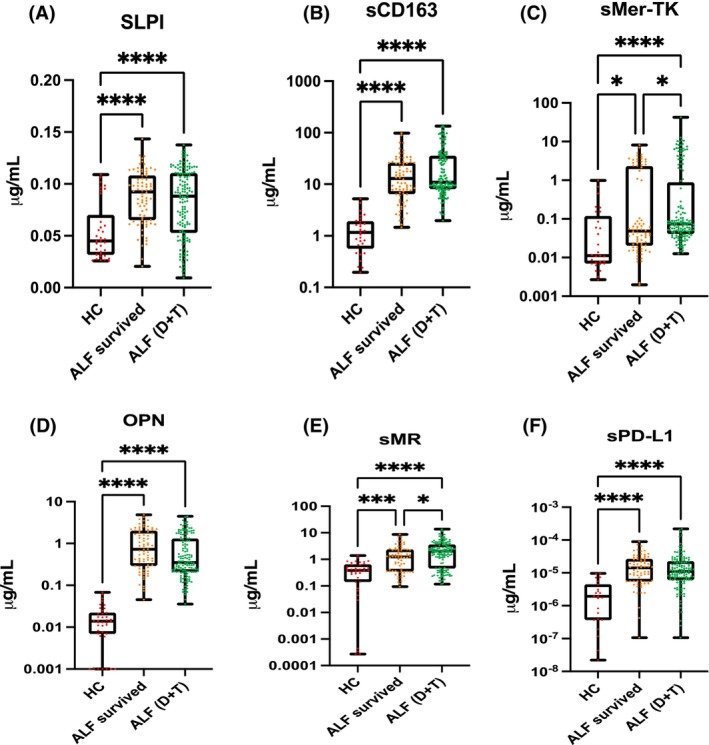
Soluble macrophage mannose receptor (sMR/sCD206) was measured from the cohort of patients with acute liver failure (ALF) in the ALF study group early (Days 1–3 following admission to hospital) and late (Day 3+) divided into those with positive or negative bacteraemia, presence or absence of infection. (A, B) Soluble macrophage mannose receptor (sMR) concentrations are higher in patient with positive bacteraemia compared to those with negative ones at early and late measurements. (D) Soluble macrophage mannose receptor (sMR) concentrations are not significantly different between those with or without presence of infection at an early time point. (E) Soluble macrophage mannose receptor (sMR) concentrations are higher in those with presence of infection compared to those without at a late time point. (C, F) Soluble macrophage mannose receptor (sMR) concentrations are significant in bacteraemia (AUROC = .753, *p* = 0.0004) and in the presence of infection (AUROC = 0.65, *p* = 0.007) in the late stage of disease. **p < 0.01, ***p < 0.001, ****p < 0.0001.

Serum SLPI concentrations were different in patients spontaneously surviving at 21 days post admission (Figure 4A) compared to patients with poor prognosis (excluding transplanted patients; ROC curve, *p* = .0010 (Figure S4)). sMerTK predicted mortality both including and excluding transplanted patients from the analysis (AUROC = .71, *p* < .0001 and AUROC = .71, *p* < .0001 respectively) (Figure S4), while PD‐L1 and CD163 were predictive only excluding the transplanted patients from the analysis (AUROC = .62, *p* = .0015 and AUROC = .68, *p* < .0001 respectively) (Figures S5 and S6). sMR was higher in those ALF who died or were transplanted compared to those who survived without transplant (Figure 4E, *p* < .05). There was significant difference between early and late samples (*p* < .0001) (Figure S7). On further analysis, OPN concentrations were higher in those patients who died compared to those who were transplanted (*p* < .0001) (data not shown). sPD‐L1 concentrations measured after Day 3 of admission were higher in those patients with ALF who died compared to those who survived (Figure S7F). There was a significant difference in sPD‐L1 concentration between early and late samples with a median respectively of 11.09 pg/mL (0–218) and 5.83 pg/mL (0–207) (*p* < .0001) (Figure S7F).

### 3.8 sCD206 and sCD163 are potential independent biomarkers of mortality

In backwards mode multivariate logistic regression modelling, later measurements of sMR/CD206 (*p* = .027) and sCD163 (*p* = .03), but not MELD, retained significance for a 21‐day spontaneous survival prediction with an area under receiver operating curve of .79 (.734–.833, *p* < .001). In MELD‐adjusted logistic regression modelling of samples after Day 3, sCD206 (*p* = .031) and sCD163 (*p* = .029) improved on raw MELD mortality prediction (AUROC 21‐day mortality with an AUROC of .81 (95% CI .73–.88, *p* < .0001) Hosmer and Lemeshow test *p* = .123).

### 3.9 External validation cohort confirmed the association of sCD206/sMR with infection in acute liver failure

**FIGURE 5:**
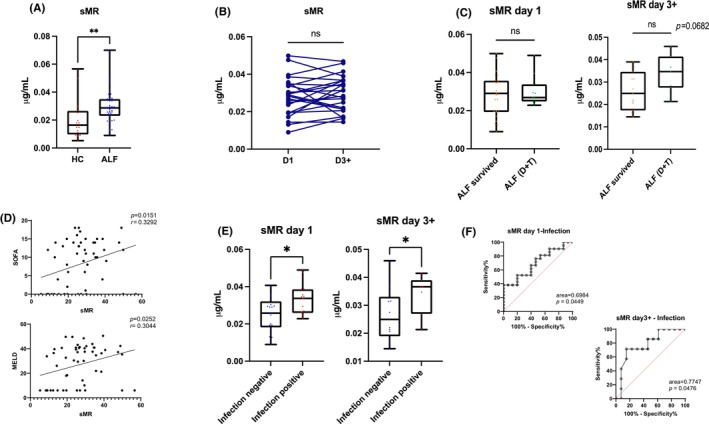
Soluble macrophage mannose receptor (sMR/sCD206) was measured in patients with acute liver failure (ALF) from the validation cohort on Days 1 and 3–7 following hospital admission. (A) Soluble macrophage mannose receptor (sMR) concentrations are higher in ALF (*n* = 40) compared to HC (*n* = 16); (B) no difference was found between admission levels and sequential samples taken between Day 3 and 7 after admission; (C) no difference in sMR was found between ALF patients spontaneously survived compared to patients deceased or transplanted (Tx) within 90 days from admission (samples from both Day 1 and Day 3+); (D) sMR directly correlates with Sequential Organ Failure Assessment (SOFA) score (top) and Model for End‐Stage Liver Disease (MELD) (bottom); (E) sMR is increased in patients with positive culture (blood, sputum or urine) both at baseline and at Day 3+; (F) ROC Curve representing sMR performance in detecting infection in ALF patients at Day 1 and Day 3+. *p < 0.05, **p < 0.01, ***p < 0.001, ****p < 0.0001.

In the validation cohort, sMR was increased in ALF patients compared to control (*p* = .00380) without a significant difference between Days 1 and 3 samples (*p* = .345) (Figure 5A,B). No difference was found in mortality according to sMR levels (Mann–Whitney *U*‐test, *p* = .78) at baseline, with a more non‐significant, trend at Day 3 (*p* = .068) (Figure 5C).Patients with a positive culture (blood, sputum or urine) had increased concentrations of sMR (Mann–Whitney *U*‐test, *p* = .0457) (Figures S11 and S12) in both early and late samples (AUROC = .71 and .77 respectively). sMR directly correlated with SOFA (*r* = .34) and MELD (*r* = .30) scores (Figure 5D).

### 3.10 CD206 is expressed on acute liver failure monocytes and is associated with sepsis and organ failure

**FIGURE 6:**
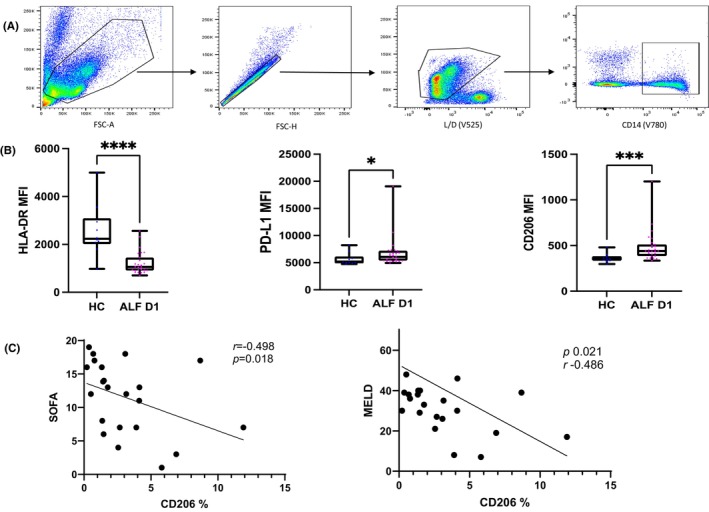
Phenotypic characterisation of CD14^+^ cells selected with flow cytometry from peripheral blood mononuclear cells (PBMCs) with acute liver failure (ALF) and healthy controls (HC). (A) Gating strategy from all cells, single cells, live/dead and CD14^+^ cells; (B) CD206, PD‐L1 and HLA‐DR expression in CD14+ cells in ALF compared to HC. (C) Surface expression of CD206 is negatively correlated with MELD/SOFA suggesting increased monocyte/macrophage shedding of these molecules with worsening disease in ALF. *p < 0.05, **p < 0.01, ***p < 0.001, ****p < 0.0001.

Expression of HLA‐DR on CD14^+^ monocytes was higher in HC compared to ALF patients as expected (*p* < .0001) (Figure 6B), while CD206 and PDL‐1 expression was higher in ALF patients (*p* < .001) (Figure 6B; Figure S14). There is a decreasing trend in CD206 expression between early (Day 1 admission) and late samples (after Day 3). The percentage of CD206‐positive cells was higher in ALF patients with sepsis compared to those without and there is a negative correlation between CD206% and SOFA score (*p* < .05, *r* = −.49) and MELD score (*p* < .05, *r* = −.48) (Figure 6C) suggesting increased monocyte shedding participates in the increases in soluble CD206 we have previously shown.

## 4 DISCUSSION

We have demonstrated that soluble markers of macrophage activation (SLPI, sMerTK, sCD163, OPN, sPD‐L1 and sMR) are increased in patients with ALF. This comprehensive analysis demonstrates prognostic potential for sCD163 and sCD206 including the novel observation that sCD206 may be a useful indicator of infection.

The dynamic nature of immune dysfunction is important in assessing this group of markers. sMerTK and sCD163 decreased after Day 3 of admission, while SLPI levels remained constant. In the early days of admission, OPN and sPD‐L1 levels were statistically higher in patients with APAP compared to AIH, DILI, indeterminate and other aetiologies (the latter only for OPN). sPD‐L1 and OPN were correlating positively with MELD at early and later time points. sCD163 improved on raw MELD mortality prediction after exclusion of OLT patients, therefore measurement of sCD163 later during admission may be a useful biomarker of mortality.

Mannose receptor (MR/CD206) is a transmembrane glycoprotein that is expressed predominantly by most tissue macrophages. It binds high mannose N‐linked glycoproteins from the blood and it interacts with pathogens that are coated with these structures. In our data, there was a significant difference between early and late samples which is explained by the progression of hepatic injury and cell damage. The soluble form(sMR) was found increased in DILI in early stages and in other aetiologies (vascular, ischaemic) later during admission.

sMR concentration was higher in patients with bacteraemia and infection in general. The same trend was seen in the validation cohort and higher CD206 expression was found on CD14^+^ monocytes from patients with sepsis and reflects immunological behaviour that will require further research in a larger cohort of patients. This finding is of clinical relevance, given that infection is one of the most important causes of death in ALF.^2^, ^20^

Moreover, the expression of surface CD206 correlates negatively with both MELD and SOFA, which may be related to shedding during the evolution of ALF or sepsis. Given that CD206 is thought to be shed via a non LPS‐dependent mechanism (in contrast to CD163),^21^ this may explain the difference in behaviour in these soluble markers. Further studies are needed to further explore the role of MR/CD206 as a biomarker of sepsis in ALF.

The other markers we investigated were upregulated in ALF but of less apparent clinical relevance. sMerTK is a tyrosine kinases receptor expressed mainly on macrophages and promotes the clearance of apoptotic cells following acute tissue injury. sMerTK was higher in DILI and indeterminate causes compared to APAP in early and late stages. Given our previous observation of increased MerTK expression on monocytes in ALF and ACLF^11^, ^22^ and the plasticity of this phenomenon with metabolic changes^23^ and sepsis it may be that this reflects the more chronic nature of DILI‐ALF over APAP‐ALF. While we are not able to define the exact date of insult or ALF initiation in all cases it is worth exploring in future work. We show that sMerTK levels fell dynamically after the third day of admission confirming the role of clearance after the initial stage of hepatic injury. However there appears little evidence for a role in sMerTK in prognostication.

sCD163 is a scavenger receptor and lineage‐specific monocyte/macrophage marker and has been associated with liver disease severity and portal hypertension.^24^, ^25^, ^26^ sCD163 levels were lower in APAP compared to other aetiologies (as vascular and ischaemic causes) when measured late in the course of the disease while Moller et al also found significantly lower levels in APAP compared to non‐APAP at Day 1.^10^ sCD163 (and sMR) has also been shown to respond to N‐acetylcysteine during therapy for APAP associated ALF.^27^

OPN is an extracellular matrix protein which is multifunctional acting in inflammation, but also in immunity and angiogenesis. It has growing interest in a wide variety of liver diseases and has been described as significantly increased in alcohol‐related hepatitis (AH) patients and it positively correlated with increasing MELD score.^28^ Our data also showed that OPN was positively correlated with MELD at both early and late stages of admission. OPN levels were higher at the start of the admission in patients with APAP, given this marker is associated with necrosis this suggests it reflects the overwhelming hepatocyte death seen in APAP‐induced ALF.

This study has several limitations. This was a retrospective analysis of prospectively collected samples, which, given the design of a cohort study, may introduce bias. Moreover, our study is not including markers of regeneration and this can be considered a limitation in the context of ALF. A recently proposed^29^ prognostic system was based on serum microRNA (miRNA) identifying a pattern of hepatic regeneration linked to recovery and outcome prediction. miRNAs can regulate macrophage activation and polarisation and macrophage‐control miRNAs have been proposed as potential new biomarker to guide selection of patients who require transplantation. We do not explore intracellular pathways related to CD206/MR but this would be of interest in future studies. Finally, while all samples were stored (−80°C) and shipped (dry ice) in temperature‐controlled environments there is the possibility that the date of recruitment could affect subsequent analyte concentrations. This will require exploration in future clinical validation procedures.

## 5 CONCLUSION

Macrophage‐activation markers are potentially useful in the prognostication of ALF patients. Measurement of sCD163 and sCD206 after Day 3 of admission are biomarkers of infection and mortality in ALF that should be further validated in future studies.

## FUNDING INFORMATION

The study was sponsored via a NIH grant DK U‐01 58369 (from NIDDK to UT Southwestern Medical Center (WL)). King's Health Partners supported the research done at King's College Hospital in London. The Medical Research Council, UK also supported this work.

## CONFLICT OF INTEREST STATEMENT

All authors have no personal or funding conflicts of interest.

## ETHICS STATEMENT

Samples from the ALFSG registry biobank (data coordinating centre at Medical University of South Carolina, Department of Public Health Sciences, Charleston, South Carolina) were used. All respective institutional review boards/health research ethics boards at participating sites (tertiary LT referral centres) within the US ALFSG approved the study's protocol. Patients from King's College Hospital were recruited as part of the ‘Immunometabolism in Sepsis, Inflammation and Liver Failure Syndromes/IMET’ (North West Haydock Research Ethics Committee Number: 19/NW/0750, IRAS Number: 244089). All research procedures were conducted according to the principles of the 1975 Declaration of Helsinki.

## PATIENT CONSENT STATEMENT

All patients or next of kin, in case of lack of capacity, signed the consent form before recruitment.

## Supporting information


Data S1.

## Data Availability

Derived data supporting the findings of this study are available from the corresponding author (MM) on reasonable request.
