# Supplementary material for: Macrophage activation markers are associated with infection and mortality in patients with acute liver failure
Source: Liver Int. 2024 Apr 8;44(8):1900–11. doi: 10.1111/liv.15928 (PMC11466005; doi:10.1111/liv.15928)
Supplement: Supplementary file 1 — Data S1. [file LIV-44-1900-s001.docx]

**Supplementary Methods**

Patients enrolled from 16 tertiary academic centres between January 1998-December 2014 who fulfilled inclusion/exclusion criteria. The study was approved by the authors’ institutional review board/health research ethics board and institutional review boards of all participating USALFSG enrolling sites and has been conducted according to the principles expressed in the 1975 Declaration of Helsinki. Written assent was obtained from the next of kin from each patient if lacking capacity to provide informed written consent due to the nature of ALF. All centres implemented monitoring and therapeutics according to institutional standards of care. Reporting of the analysis of this study complies to the STROBE Guidelines for reporting case-control studies^1^.

Operational Definitions

For the purpose of this study, ALF was defined using the following criteria: (1) international normalized ratio (INR) $\geq$1.5, (2) HE of any grade (West Haven Criteria), (3) illness onset less than 26 weeks from hepatic insult, and (4) absence of existing cirrhosis. The King’s College Criteria (KCC) qualify poor prognostic signs in ALF. In APAP-ALF, KCC is defined as either (1) arterial pH $<$7.3, or (2) all three of i) INR $>$6.5, ii) creatinine $>$300 µmol/L (3.4 mg/dL), and iii) the presence of grade 3/4 HE. The Acute Liver Failure Study Group Prognostic Index (ALFSG-PI) is an internally-validated mathematical model that predicts 21-day TFS of patients with ALF using hospital admission data and has been previously described^2^. The model for end stage liver disease (MELD) is calculated as follows: [3.78*ln (bilirubin in mg/dL) + 11.2*ln (INR) + 9.57*ln (creatinine in mg/dL) + 6.43]; a serum creatinine value of 354 µmol/L (4 mg/dL) is substituted for dialyzed patients. RRT included both intermittent hemodialysis (IHD) and continuous hemofiltration (CRRT). Patients receiving CRRT and IHD during days 1-7 were coded accordingly. The use of RRT within the US ALFSG is not standardized; thus, modality, replacement fluid, anticoagulation, dose, and indications for initiation and cessation of therapy were based on intensivist judgement at the enrolling center.

PBMC Isolation

Heparinised blood was diluted 1:1 v/v with phosphate buffer solution (PBS, GibcoTM, Thermofisher, USA) in 50mL tube(s) and used to isolate PBMC by density gradient centrifugation using Ficoll-Paque Plus (GE Healthcare, UK) at 1:3 v/v. Centrifuge: RCF 800xg for 20 min at room temperature with brake off. PBMCs were collected and resuspended with PBS and then centrifuged twice: RCF 800xg for 10min at room temperature with the brake on. The cell pellet was re-suspended in the desired volume according to following use destination, i.e., 1mL of complete medium for culture including RPMI 1640 medium (Gibco, Thermofisher USA)+ 10% Fetal Bovine Serum (FBS, Thermofisher) + 1% Penstrep (Penicillin-Streptomycin 10,000 U/mL, Gibco, Thermofisher, USA), or 500 μL of RPMI and transferred into 1ml cryovials containing 500 μL of freezing mix (20% Dimethylsulfoxide (DMSO) + 80% FBS to obtain a final concentration of 10% DMSO, 40% FBS, 50% medium), and then freeze/stored at -80°C for future phenotyping.

Monoclonal antibodies (CD14, CD16, HLA-DR, MerTK, CD163, CD206, PD-1) were used to phenotye monocytes with flow cytometry. Results were shown as percentage (%) and mean fluorescence intensity (MFI). BD LSRFortessa™ cell analyzer (BD Biosciences) was used to acquire data, FCS files were gated manually to single, live, CD14+ cells using FlowJo™ v10 (Becton Dickinson & Company). PhenoGraph and t-SNE analysis were performed using the R package ‘CyTOFkit’^3^. PhenoGraph identified the k-nearest neighbors using Euclidean distance, Jaccard coefficient was used to calculate similarities and portioning of the dataset to detect communities with optimal modularity was performed using Louvain algorithm (13 groups). Default parameters (interactions=1000 perplexity=30) were set form performing t-SNE.

**Supplementary Table 1. Clinical variables and macrophage activation markers from the cohort of patients with ALF in the acute liver failure study group. Categorical variables are given as n(%) and continuous as median (range).**

| Variables | Total | ALF Died | ALF Alive | p - value |
| --- | --- | --- | --- | --- |
| Gender male | 79/224 (36) | 28/76 (37) | 23/74 (31) | 0.456 |
| Age (years) | 42 (17-81) | 43 (18-81) | 41 (17-78) | 0.744 |
| Aetiology- APAP (Acetaminophen) | 60/224 (27) | 26/76 (34) | 32/76 (42) | 0.261 |
| Aetiology- AIH (autoimmune hepatitis) | 15/224 (7) | 1/76 (1) | 0 (0) |  |
| Aetiology- DILI (Drug induced liver injury) | 87/224 (39) | 25/76 (33) | 25/76 (33) |  |
| Aetiology- Indeterminate | 46/224 (20) | 13/76 (17) | 15/76 (20) |  |
| Aetiology-Other (Vascular/hypoxic) | 16/224 (7) | 11/76 (15) | 4/76 (5) |  |
| Hb (g/dL) | 11 (6-17) | 10.4 (5.8-17) | 10 (6-15) | 0.514 |
| WBC (10^9/L) | 9.6 (0.2-35) | 10.3 (0.2-30) | 8.8 (1.5-35)c | 0.117 |
| PLT (10^9/L) | 115 (6-386) | 110 (14-386) | 110 (6-335) | 0.998 |
| ALT (IU/L) | 900 (10-18079) | 934 (10-14410) | 1455 (24-14725) | 0.619 |
| AST (IU/L) | 802 (37-22725) | 1011 (37-22725) | 1070 (51- 19632) | 0.934 |
| Bilirubin (mg/dL) | 14.4 (0.5-63) | 14 (1-53) | 7.3 (0.5-48.5) | 0.009 |
| Creatinine (mg/dL) | 1.4 (0.34-8.3) | 2.1 (0.5-5.5) | 1.4 (0.4-8.3) | 0.119 |
| Na (mmol/L) | 138 (122-164) | 140 (123-155) | 137 (122-164) | 0.080 |
| Phosphate (mmol/L) | 3 (1-10) | 3 (1-10) | 2.75 (1-7) | 0.315 |
| PH | 7.42 (7-8) | 7.40 (7.0-8.0) | 7.41 (7-8) | 0.444 |
| Fi02 (%) | 40 (21-100) | 42.5 (20-100) | 30 (0-100) | 0.031 |
| Venous NH3 | 85 (28-918) | 109 (35-918) | 73 (29-218) | 0.002 |
| INR (Ratio) | 2.6 (1-16) | 3.4 (1-15) | 2.1 (0.9-8) | 0.001 |
| Lactate | 4 (0-20) | 7.6 (2-20) | 2.6 (1-7) | <0.001 |
| RTT (Renal Replacement Therapy) | 48/222 (22) | 20/76 (26) | 22/75 (29) | 0.679 |
| Sepsis | 29/217 (13) | 11/74 (15) | 11/74 (15) | 1.000 |
| High grade HE (hepatic encephalopathy) | 95/212 (45) | 49/71 (69) | 31/74 (42) | 0.001 |
| ICP monitor | 16/203 (8) | 5/71 (7) | 8/74 (11) | 0.427 |
| MELD score | 32 (8-59) | 36 (  22-59) | 30 (8-50) | <0.001 |
| KCC (Kings College Criteria) yes/no | 57/200 (28.5)/ 143/200 (71.5) | 20/70 (29)/ 50/70 (71) | 12/65 (18.5)/ 53/65 (81.5) | 0.168 |
| SLPI (10^-3^µg/ml)  76 D; 76 S | 89.87  (9.46-143.3) | 109.9  (9.46-137.5) | 92.34  (20.52-143.3) | 0.016 |
| sCD163 (µg/ml)  76 D; 76 S | 11.26  (1.46  -134) | 10.60  (2.59-134.6) | 12.93 (14.65- 98.39) | 0.344 |
| sMerTK (10^-3^µg /ml)  76 D; 76 S | 63.48  (1.96- 42183.5) | 75.35  (12.49 - 42183.5) | 48.38  (1.96 - 8147.6) | 0.008 |
| OPN (10^-3^µg /ml)  76 D; 76 S | 438.88  (35.71-  4842.13) | 927.15  (69.56- 4461.79) | 728.17  (45.46-4842.13) | 0.570 |
| sMR (10^-3^µg /ml)  74 D; 71 S | 1610.70  (93.65-  13727.5) | 1590.06  (151.60 - 13727.5) | 1236.55 (  93.65-  8671.67) | 0.105 |
| sPDL1 (10^-6^µg/ml)  76 D; 76 S | 11.1 (0-218) | 16.4 (0-218) | 13.0 (0-89) | 0.094 |

**SUPPLEMENTARY FIGURES**

**
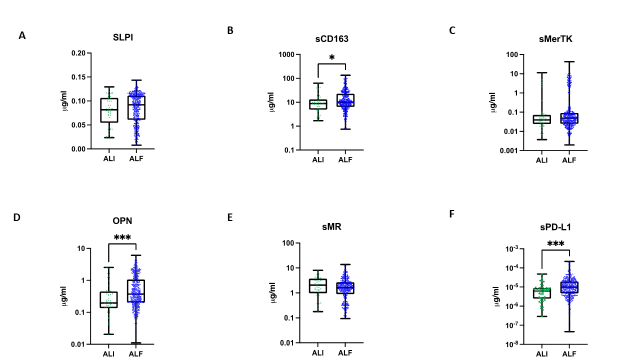
**

**Suppl.figure 1: Macrophage activation markers in the serum of patients with acute liver injury (ALI) and acute liver failure (ALF) measured by enzyme linked immune-absorbent assay (ELISA). B, D, F) soluble cluster of differention 163 (sCD163), Osteopontin (OPN) and soluble programmed death ligand 1 (sPD-L1) concentrations are higher in ALF compared to ALI patients**. A, C, E) There are no significant differences between ALI and ALF patients for Secretoty leukocyte protease inhibitor (SLPI), soluble Mer Tyrosine Kinase (sMerTK) and soluble macrophage mannose receptor (sMR).


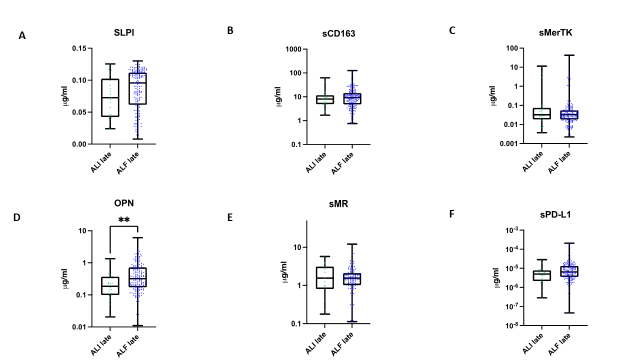


**Suppl.figure 2: Macrophage activation markers in the serum of patients with acute liver injury (ALI) and acute liver failure (ALF) measured by enzyme linked immune-absorbent assay (ELISA) late (day 3+) during admission**. D) Osteopontin (OPN) concentrations are higher in ALF compared to ALI at a late time point. A, B, C, E, F) Secretory leukocyte protease inhibitor (SLPI), soluble cluster of differentiation 163 (sCD163), soluble Mer Tyrosine Kinase (sMerTK), soluble macrophage manose receptor (sMR) and soluble programmed death ligand 1 (sPD-L1) concentrations are not statistically different between ALI and ALF late during the admission.


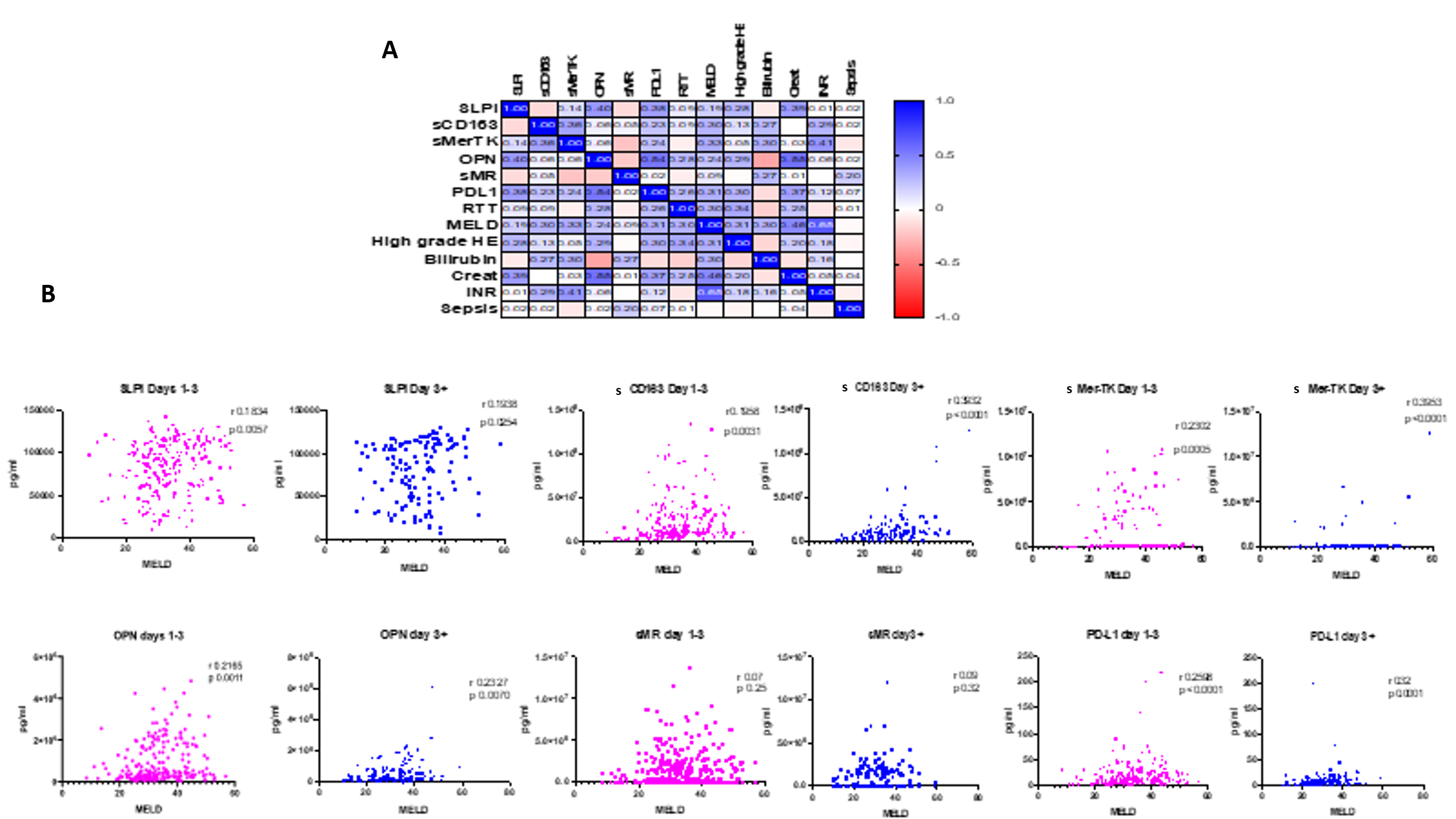


**Suppl. figure 3: A) Macrophage activation markers in the serum of patients with acute liver failure (ALF) measured by enzyme linked immune-absorbent assay (ELISA) correlated with clinical features. B) Macrophage activation markers correlated with MELD early (day 1-3) and late (day 3+) in the course of disease**. A) Correlation matrix between six macrophage activation markers and clinical features as renal replacement therapy (RTT), Model for end stage liver disease (MELD), high grade hepatic encephalopathy (HE), bilirubin (bili), creatinine (Creat), INR, sepsis. Secretoty leukocyte protease inhibitor (SLPI), Osteopontin (OPN), soluble programmed death ligand 1 (sPDL1) correlate with Creatinine. Soluble cluster of differention 163 (sCD163), soluble mer tyrosine kinase (sMerTK), OPN, sPDL1 correlate with MELD. sCD163, sMerTK, soluble macrophage manose receptor (sMR) correlate with Bilirubin. sCD163 and sMerTK correlate with INR. OPN and sPDL1 correlate with RTT. OPN and sPDL1 correlate with high grade HE. sMR also correlate with sepsis. (B) Correlation between six macrophage activation markers and MELD early (day 1-3) following admission to hospital) and late (day 3+) in the course of disease. Most of the markers are positively correlated with MELD at both early and late time points, SLPI (r=0.1834, p 0.0057; r= 0.1938, p 0.0254), sCD163 (r=0.1958, p 0.0031; r=0.3932, p<0.0001), sMerTK (r= 0.2302, p= 0.0005; r=0.3953, p<0.0001), OPN (r=0.216, p=0.0011; r=0.232, p=0.007) and sPDL1 (r= 0.2598, p< 0.0001; r=0.32, p= 0.0001). sMR has no significant correlation with MELD at early (r= 0.07, p 0.25) or late (r 0.09, p= 0.32) stages.

**Suppl.figure 4-6: Receiver operating characteristic (ROC) curves for each of the measured markers including (tx in) and excluding (tx out) the transplanted patients. Six molecules were measured from the cohort of patients with ALF in the acute liver failure study group early (day 1-3 following admission to hospital) and late (day 3+) in the course of disease.**


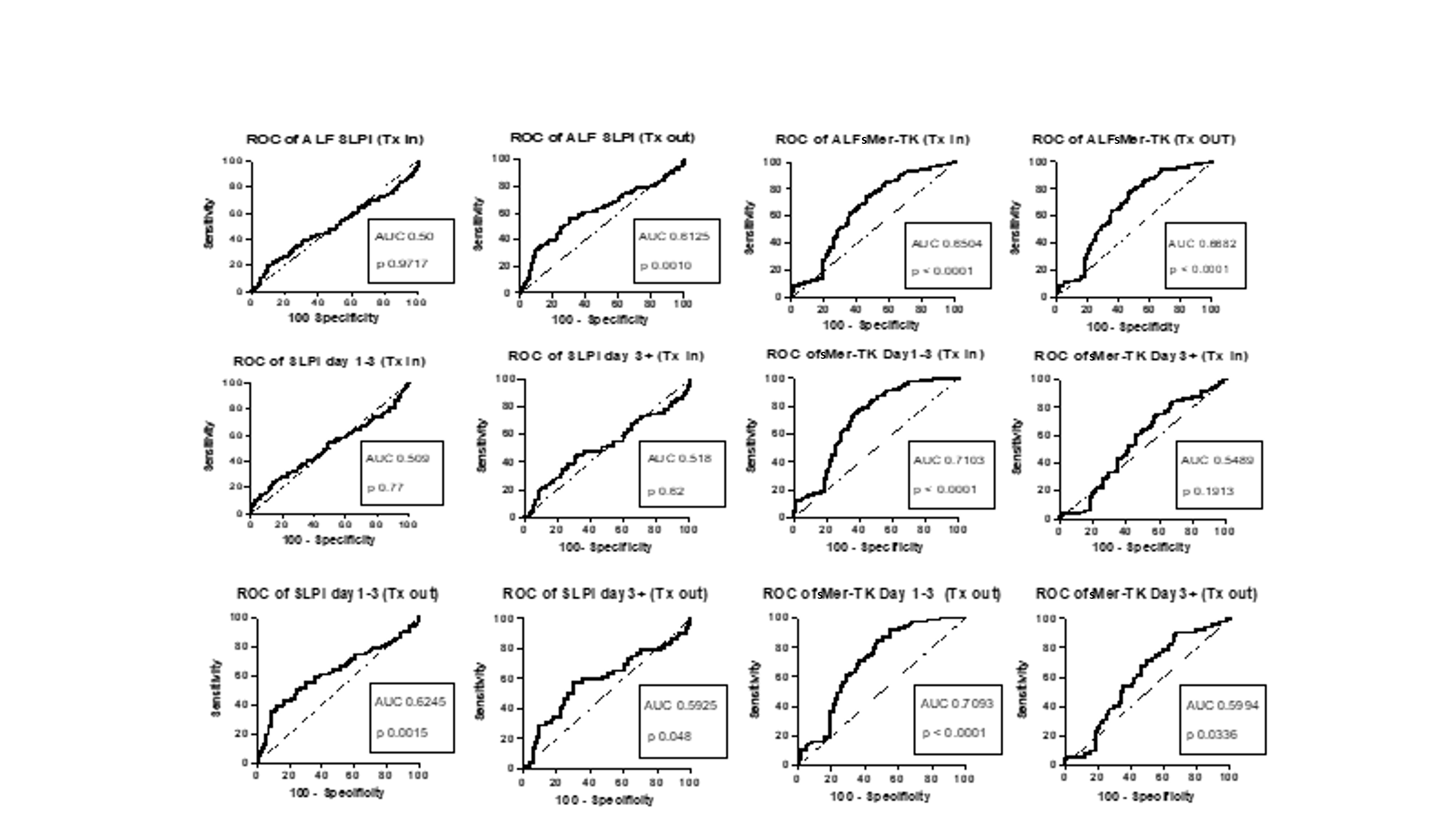


**4:** Secretory leukocyte peptidase inhibitor (SLPI) concentrations are significant only when excluding transplants (AUROC= 0.6125, p= 0.0010) and also when split in early (AUROC= 0.6245, p= 0.0015) and late (AUROC= 0.5926, p= 0.048) groups. soluble mer tyrosine kinase (sMerTK) concentrations are significant including (AUROC= 0.6504, p< 0.0001) and excluding (AUROC= 0.6682, p< 0.0001) transplants.


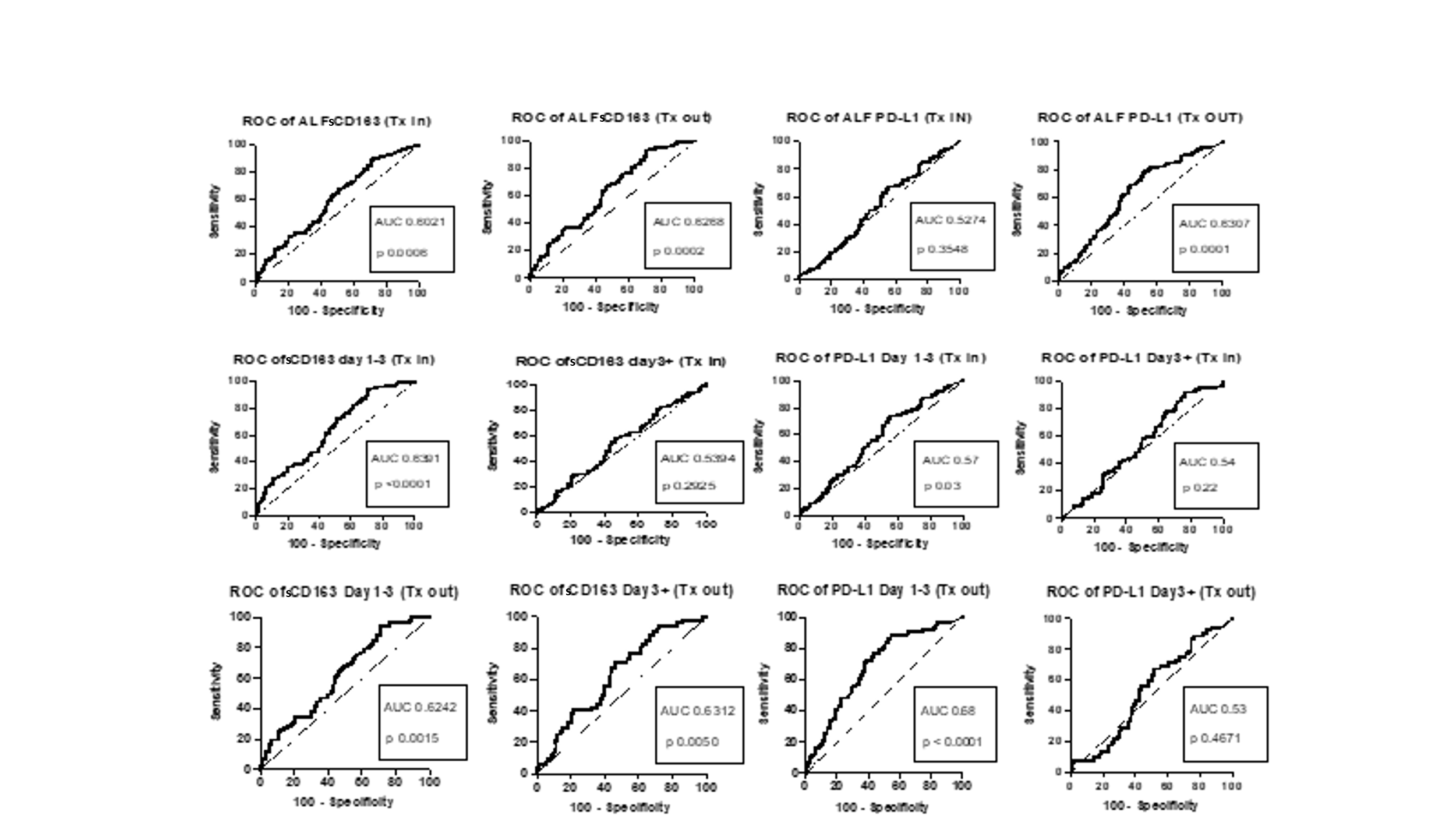


**5:** Soluble cluster of differention 163 (sCD163) concentrations are significant when including and excluding transplants (respectively AUROC= 0.6021, p= 0.0006; AUROC= 0.6268, p= 0.0002). The significance is maintained in the early group with and without transplants (AUROC= 0.6391, p< 0.0001; AUROC= 0.6242, p= 0.0015) and in the late group only excluding transplants (AUROC= 0.6312, p= 0.0050). soluble programmed death ligand 1 (sPD-L1) concentrations are significant only in the early group both when including and excluding transplants (respectively AUROC= 0.57, p= 0.03; AUROC= 0.68, p< 0.0001).


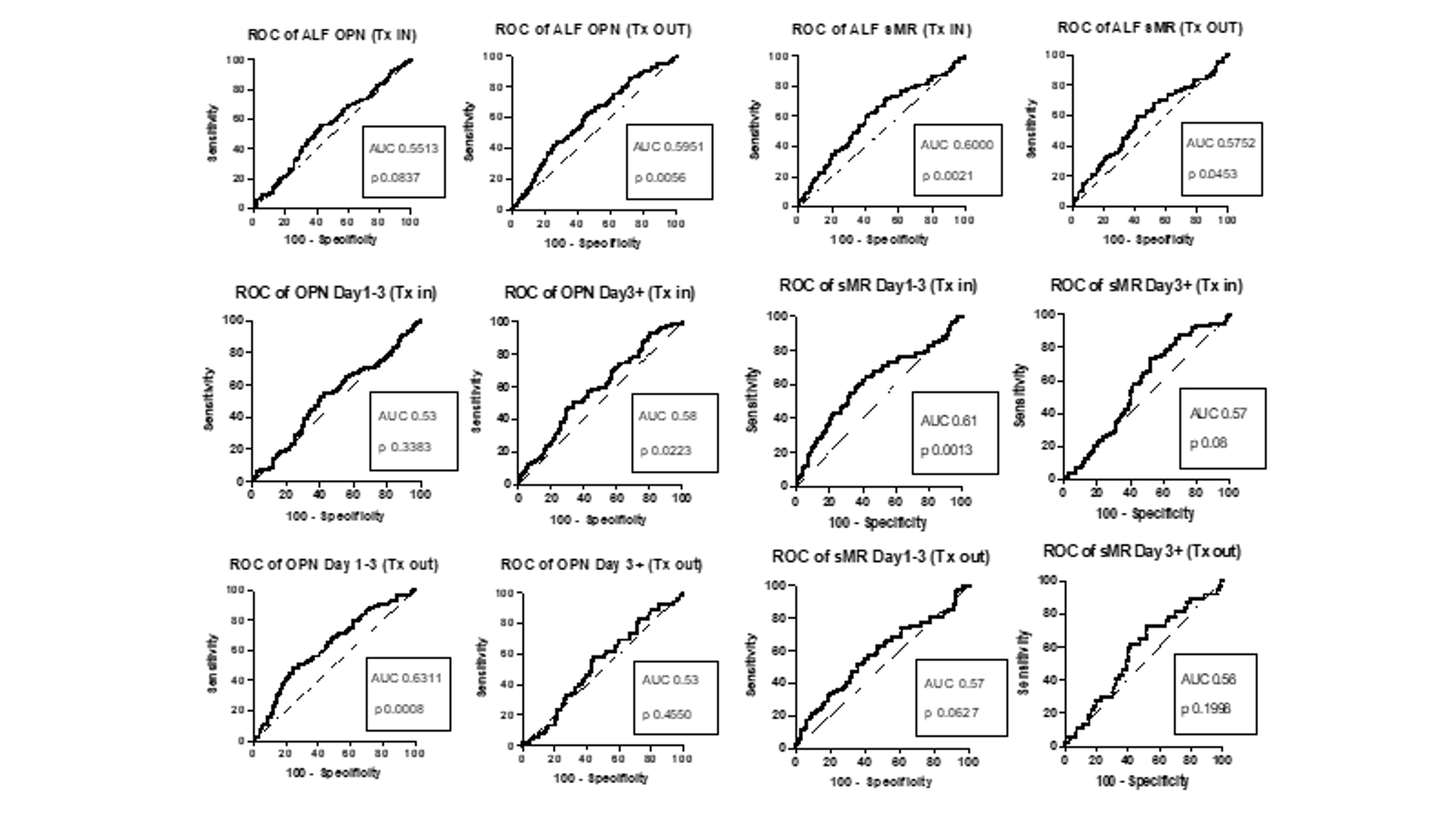


**6:** Osteopontin (OPN) concentration is significant the late group when including transplants (AUROC= 0.58, p= 0.0223) and in the early group when excluding transplants (AUROC= 0.6311, p= 0.0008). soluble macrophage manose receptor (sMR) concentration is significant only when including transplants (AUROC= 0.6000, p= 0.0021) and in the early group (AUROC= 0.61, p= 0.0013).


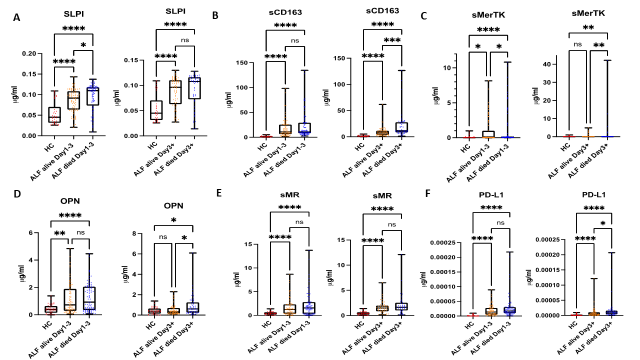


Suppl. figure 7: **Macrophage activation markers in the serum of patients with acute liver failure (ALF) and healthy controls (HC) measured by enzyme linked immune-absorbent assay (ELISA) early (day 1-3) and late (day 3+) during admission.** A) Secretory leukocyte protease inhibitor (SLPI) concentrations are higher in ALF compared to HC and higher in those ALF patients who died compared to survivors at an early time point, B) soluble cluster of differention 163 (sCD163) concentrations are higher in ALF compared to HC and later measurement discriminates those who died from survivors, C) soluble Mer Tyrosine Kinase (sMerTK) concentrations are higher in ALF compared to HC and early measurement in ALF patients show higher concentrations in those who died compared to survivors, D) Osteopontin (OPN) is higher in ALF patients compare to controls and survivors show reduced concentrations to non surivivors at both time points, E) soluble macrophage manose receptor (sMR) is higher in patients with ALF compared to HC but does not discriminate survivors from non survivors, F) soluble programmed death ligand 1 (sPD-L1) has a higher concentration in serum of ALF patients compared to HC and is higher in those ALF patients who died when measured later in the disease course.
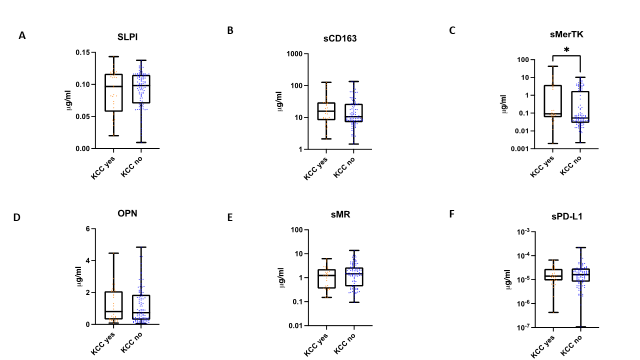


**Suppl. figure 8: Macrophage activation markers in the serum of patients with acute liver failure (ALF) measured by enzyme linked immune-absorbent assay (ELISA). Six molecules were measured from the cohort of patients with ALF in the acute liver failure study group and King’s College Criteria (KCC) were considered.** A, B, D, E, F) Secretory leukocyte peptidase inhibitor (SLPI), soluble cluster of differentiation 163 (sCD163), Osteopontin (OPN), soluble macrophage manose receptor (sMR) and soluble programmed death ligand 1 (sPD-L1) concentrations are not significantly different in those who met or did not KCC. C) Soluble mer tyrosine kinase (sMerTK) concentrations are higher in those who met KCC (respectively p<0.05; p< 0.0001) compared to those patients who did not.


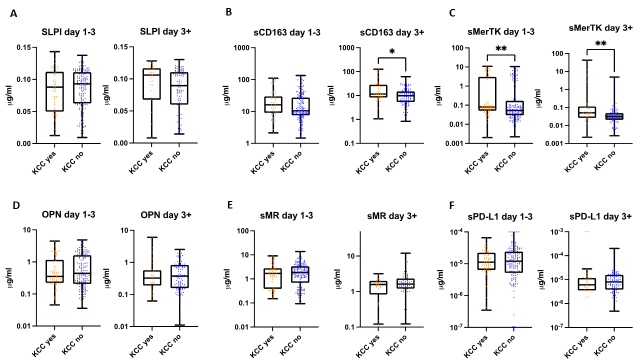


**Suppl figure 9: Macrophage activation markers in the serum of patients with acute liver failure (ALF) measured by enzyme linked immune-absorbent assay (ELISA) early (day 1-3) and late (day3+) during admission considering King’s College Criteria (KCC).** A, D, E, F) Secretoty leukocyte protease inhibitor (SLPI), Osteopontin (OPN), soluble macrophage manose receptor (sMR), soluble programmed death ligand 1 (sPD-L1) concentrations are not significally different when meeting or not KCC at early or later stage. B) soluble cluster of differention 163 (sCD163) concentrations are higher when meeting KCC at later stage of disease. C) soluble mer tyrosine kinase (sMerTK) concentrations are higher when meeting KCC at early and late stage of disease.


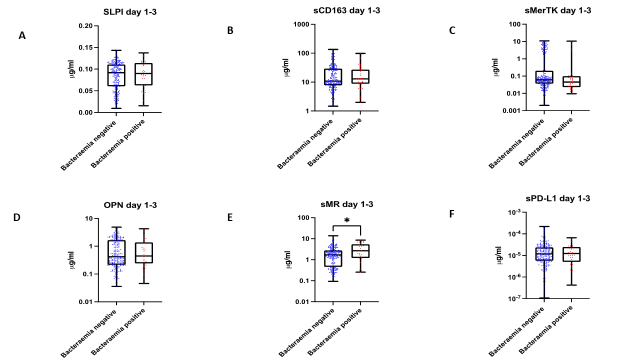


**Suppl. figure 10: Macrophage activation markers in the serum of patients with acute liver failure (ALF) measured by enzyme linked immune-absorbent assay (ELISA). Six molecules were measured from the cohort of patients with ALF in the acute liver failure study group early (day 1-3 following admission to hospital) divided into those with negative or positive bacteraemia**.

A, B, C, D, F) Secretoty leukocyte peptidase inhibitor (SLPI), soluble cluster of differention 163 (sCD163), Soluble mer tyrosine kinase (sMerTK), Osteopontin (OPN) and soluble programmed death ligand 1 (sPD-L1) concentrations are not significantly different between those with positive and negative bacteraemia. E) Soluble macrophage manose receptor (sMR) concentrations are higher in those with positive bacteraemia compared with those with negative ones.


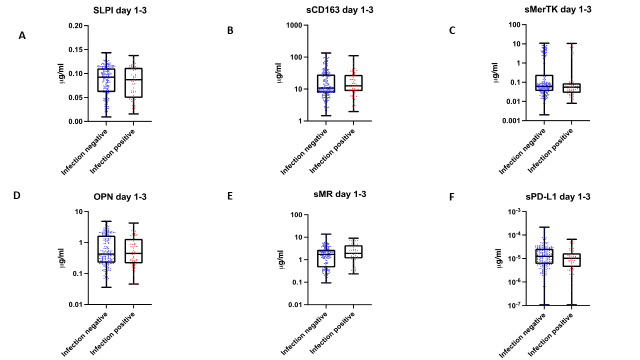


**Suppl. figure 11: Macrophage activation markers in the serum of patients with acute liver failure (ALF) measured by enzyme linked immune-absorbent assay (ELISA). Six molecules were measured from the cohort of patients with ALF in the acute liver failure study group early (day 1-3 following admission to hospital) divided into those with presence or absence of infection.**

A, B, C, D, E, F) Secretory leukocyte peptidase inhibitor (SLPI), soluble cluster of differentiation 163 (sCD163), Soluble mer tyrosine kinase (sMerTK), Osteopontin (OPN), Soluble macrophage manose receptor (sMR) and soluble programmed death ligand 1 (sPD-L1) concentrations are not significantly different between those with or without presence of infection.


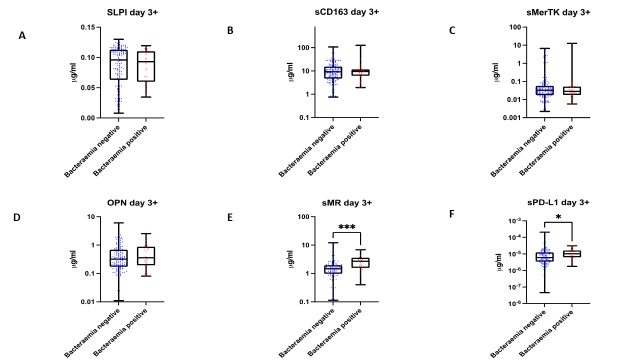


**Suppl. figure 12: Macrophage activation markers in the serum of patients with acute liver failure (ALF) measured by enzyme linked immune-absorbent assay (ELISA). Six molecules were measured from the cohort of patients with ALF in the acute liver failure study group late (day 3+) in the course of disease divided into those with negative or positive bacteraemia**. A, B, C, D) Secretory leukocyte peptidase inhibitor (SLPI), soluble cluster of differentiation 163 (sCD163), Soluble mer tyrosine kinase (sMerTK) and Osteopontin (OPN) concentrations are not significantly different between those with positive and negative bacteraemia.

E, F) Soluble macrophage manose receptor (sMR/sCD206) and soluble programmed death ligand 1 (sPD-L1) concentrations are higher in those with positive bacteraemia compared with those with negative ones.


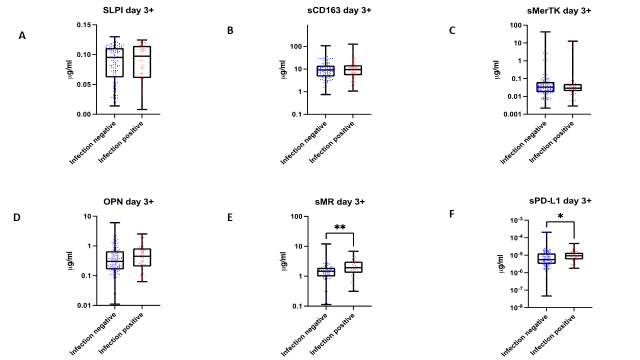


**Suppl. figure 13: Macrophage activation markers in the serum of patients with acute liver failure (ALF) measured by enzyme linked immune-absorbent assay (ELISA). Six molecules were measured from the cohort of patients with ALF in the acute liver failure study group late (day 3+) in the course of disease divided into those with presence or absence of infection**. A, B, C, D) Secretory leukocyte peptidase inhibitor (SLPI), soluble cluster of differentiation 163 (sCD163), Soluble mer tyrosine kinase (sMerTK) and Osteopontin (OPN) concentrations are not significantly different between those with or without presence of infection. E, F) Soluble macrophage manose receptor (sMR) and soluble programmed death ligand 1 (sPD-L1) concentrations are higher in those with presence of infection compared to those without it.


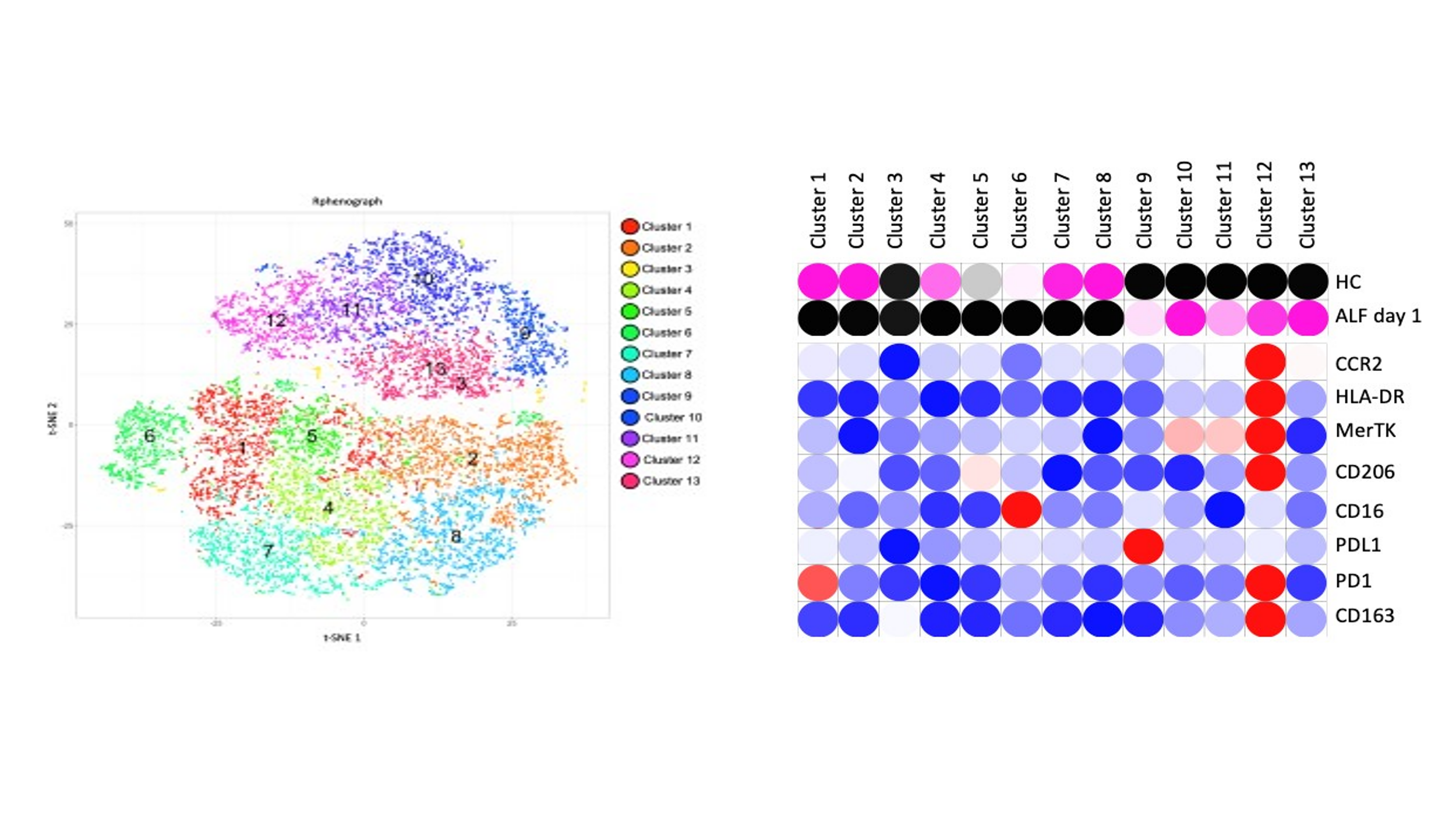
**Suppl. Figure 14**: Surface expression on CD14+ monocytes in acute liver failure (ALF) and healthy controls (HC) measured by Flow Cytometry and analysed via mass cytometry CytofKit demonstrating clear discrimination on 12 clusters driven by HLADR, CD206, PD1 and CD163 expression (p<0.05)

**Supplementary References**

1. von Elm E, Altman DG, Egger M, et al. Strengthening the Reporting of Observational Studies in Epidemiology (STROBE) statement: guidelines for reporting observational studies. BMJ. 2007;335(7624):806-8.

2. Tavabie OD, Karvellas CJ, Salehi S, et al. A novel microRNA-based prognostic model outperforms standard prognostic models in patients with acetaminophen-induced acute liver failure. J Hepatol. 2021;75(2):424-34. Epub 20210412.

3. Chen H, Lau MC, Wong MT, et al. Cytofkit: A Bioconductor Package for an Integrated Mass Cytometry Data Analysis Pipeline. PLoS Comput Biol. 2016;12(9):e1005112. Epub 20160923.
